# Surfactant-Enhanced Hybridization Capture for Direct Sequence-Specific RNA Extraction from Plasma and Whole Blood

**DOI:** 10.64898/2026.10.06.757078

**Published:** 2026-10-12

**Authors:** Wookyeom Kim, Shane Gilligan-Steinberg, Enos C. Kline, Ingrid Beck, Lisa M Frenkel, Barry Lutz

## Abstract

Direct recovery of low-abundance RNA from complex biological samples remains a major challenge for ultrasensitive detection. Sequence-specific hybridization capture enables selective enrichment, but recovery from crude samples is compromised by RNA degradation, protein interference, RNA secondary structure, and target sequence variation. Here, we address these limitations with a sodium dodecyl sulfate (SDS)-based formulation that not only lyses virions and protects RNA, but also directly accelerates hybridization kinetics, substantially reducing the need for thermal denaturation. This enabled direct RNA capture from crude plasma or whole blood with near-quantitative recovery, outperforming a widely used silica-based kit at low input. Without centrifugation or plasma separation, we detected HIV RNA by RT-qPCR at 25 copies/mL directly from whole blood. We further developed a free energy (ΔG)-guided algorithm for mutation-robust probe design, achieving >85% mean recovery across HIV-1 subtypes with a single probe. This work opens low-abundance RNA biomarkers in crude biofluids to ultrasensitive detection, addressing a major barrier to early diagnosis.

## Full Text Availability

The license terms selected by the author(s) for this preprint version do not permit archiving in PMC. The full text is available from the preprint server.

